# NTG-101: A Novel Molecular Therapy that Halts the Progression of Degenerative Disc Disease

**DOI:** 10.1038/s41598-018-35011-4

**Published:** 2018-11-14

**Authors:** Ajay Matta, Muhammad Zia Karim, Hoda Gerami, Peter Jun, Martha Funabashi, Greg Kawchuk, Alyssa Goldstein, Warren Foltz, Marshall Sussman, Bjorn C. Eek, W. Mark Erwin

**Affiliations:** 1 Notogen Inc., Toronto, Ontario, Canada; 2 University of Alberta, Edmonton, Alberta Canada; 3 University Health Network, Toronto, Ontario, Canada; 4 Private Practice, Irvine, California USA; 5 Department of Surgery, University of Toronto, Toronto, Ontario, Canada; 6 Canadian Memorial Chiropractic College, Toronto, Ontario Canada

## Abstract

The tremendous cost, pain and disability associated with degenerative disc disease (DDD) makes the development of a biological agent that can mitigate the course of DDD, a critical unmet need. We have identified and reported that a single injection of a combination of recombinant human (rh) Transforming growth factor beta 1 (TGF-β1) and Connective tissue growth factor (CTGF) proteins into the injured intervertebral disc (IVD) nucleus pulposus (NP) can mediate DDD in a pre-clinical rodent model. In this study, we developed and evaluated the efficacy of a novel molecular therapy (NTG-101) containing rhTGF-β1 and rhCTGF proteins suspended in an excipient solution using *in vivo* models of DDD including rat-tail and chondrodystrophic (CD) canines. Needle puncture injury in CD-canine NPs resulted in loss of hydration, disc height and showed radiographic evidence of DDD like humans. However, NTG-101-injected IVDs maintained disc height and demonstrated retention of viscoelastic properties as compared to IVDs injected with phosphate buffer saline (PBS, 1X, pH = 7.2). In addition, a single intra-discal injection of NTG-101 into the injured IVD-NPs resulted in sustained expression of healthy extra-cellular matrix (ECM) proteins (aggrecan, collagen 2A1) and reduced expression of inflammation associated proteins and molecules (IL-1β, IL-6, IL-8, MMP-13, Cox-2 and PGE2) as compared to vehicle controls. In conclusion, we demonstrated that a single intra-discal injection of the novel formulation, NTG-101 confers a robust anti-inflammatory, anti-catabolic and pro-anabolic effects in pre-clinical models of DDD thereby restoring homeostasis. These findings suggest the therapeutic potential of NTG-101 for clinical use.

## Introduction

Degenerative disc disease (DDD) results in significant disability, tremendous expense and the highest number of disability-adjusted life years globally^1–3^. Costs to the healthcare system associated with DDD-related disability have remained fairly consistent for a number of years (1991–2007) with estimates that range between USD $50–100 billion/annum^3–5^. In fact, a direct correlation with the severity of DDD on MRI and back pain has been reported in a large population based study of 1043 individuals^6^. Numerous attempts to treat DDD with biological therapy have not yet achieved the desired result^7–10^. Given the tremendous costs, pain and disability associated with DDD and the current lack of any disease modifying treatment, a biological therapy that could mediate the progression of DDD or even confer a regenerative effect would revolutionize the approach to DDD. With respect to the development of an effective molecular therapeutic, chondrodystrophic (CD) canines that have a propensity to develop DDD, and the non-chondrodystrophic (NCD) canines that are protected from developing DDD, offer a unique platform for investigation. We and others have extensively reported on the anti-degenerative effects of Notochordal Cell Conditioned Medium (NCCM) obtained from the notochordal cell-rich intervertebral disc nucleus pulposus (IVD-NP) of the NCD-canine^11–20^. Recently, we injected NCCM into the needle puncture injury induced degenerative rat tail IVD-NP and demonstrated that soluble factors present in NCCM can mitigate the progression of DDD^18^. Amongst the thousands of soluble proteins contained within NCCM, we identified and showed that a single injection containing rhTGF-β1 and rhCTGF recapitulated the anti-degenerative and pro-anabolic effects of a single injection of NCCM in rat-tail needle puncture model of DDD^18^.

In the current study, we evaluated the efficacy of a novel, molecular therapeutic ‘NTG-101’ containing a combination of rhTGF-β1 and rhCTGF proteins suspended in an excipient solution in *in vivo* models of DDD including rat-tail NP and CD-canine NPs. Our results demonstrated a single intra-discal injection of NTG-101 confers anti-degenerative effects leading to reduction in expression of pro-inflammatory cytokines including interleukin-1 beta (IL-1β), interleukin–6 (IL-6), interleukin–8 (IL-8), ECM degrading enzymes (MMP-13), cyclooxygenase-2 (Cox-2) while inducing pro-anabolic effects upon the IVD - NP restoring expression of healthy ECM proteins (aggrecan, collagen 2A1). In addition, we also observed the maintenance of disc height and biomechanical characteristics in IVDs injected with NTG-101 as compared to vehicle controls in CD-canines. These findings suggested the therapeutic potential of NTG-101 for clinical use in future.

## Materials and Methods

### Animals and Ethics Statement

All animals (rats/CD-canines) were obtained in collaboration with a licensed animal facility and all experiments were conducted in Canadian Council on Animal Care (CCAC) accredited facilities (University Health Network, Toronto). All experimental protocols were carried out in accordance with CCAC policies and guidelines and approved by the Institutional Animal Care Committee of University Health Network (UHN), Toronto, Ontario, Canada.

### Cell Viability, Cell Proliferation Assay and Gene Expression Analysis

Human degenerative disc nucleus pulposus tissues were obtained from patients (n = 8) undergoing discectomy or fusion surgery at Toronto Western Hospital, University Health Network (UHN), Toronto with all cases obtained in accordance with the guidelines approved by the Research Ethics Board, Toronto Western Hospital, UHN, Toronto. Nucleus pulposus (NP) tissue was enzymatically digested and cells were cultured within a hypoxic incubator (NuAire, MN, USA) as described earlier^17,18^. Thereafter the cells were either cultured in serum free ADMEM (no treatment controls) or treated with rhIL-1β (10 ng/ml), tumor necrosis factor alpha (rhTNFα, 50 ng/ml), or a combination of rhCTGF (100 ng/ml) + rhTGF-β1 (10 ng/ml) proteins for 24–72 hrs under hypoxic conditions to determine the effect of the treatment on cell viability, proliferation and ECM synthesis^17,18^. For details, see Supplementary data.

### Intra-Discal Injection of NTG-101 in Pre-Clinical Rodent Model of DDD

We used our established rat-tail needle puncture injury model of DDD using 12-week old female Wistar rats (n = 27, Charles River Laboratories Inc.) as described earlier^18^. IVD needle puncture injury was performed in 5-caudal discs per animal using fluoroscopic image guidance as described earlier^18^. Four weeks post injury, animals were randomized into 3 groups (including 9 animals per group), and the injured discs were injected with 8.0 µl of vehicle (i.e. phosphate buffer saline, PBS, 1X, pH = 7.2) or excipient solution (ES) or NTG-101 using a 32 G needle under fluoroscopic guidance. At the end of the experiment (10 weeks post-injury), the animals were humanely euthanized. At least one caudal IVD per animal was fixed in formalin as a representative for histological analysis. Age-matched (22-week-old) healthy IVDs were obtained from rat tail IVDs that served as uninjured, healthy controls.

### Intra-Discal Injection of NTG-101 or Vehicle (PBS, 1X) in Pre-Clinical CD-Canine Model of DDD

All animals (n = 16) were obtained from a licensed animal testing facility (Kingfisher International Inc., Stouffville, Ontario, Canada). Briefly, the disruptive needle puncture injury was performed in 3-non-contiguous lumbar IVD-NPs at levels (L1/2, L3/4 and L5/6) by a clinical Veterinarian in 3-year old chondrodystrophic (CD) canines (age matched) using fluoroscopic guidance. Four weeks post-injury animals were randomized and were administered with a single intra-discal injection of 350.0 µl of either vehicle (Group 1, n = 6) or NTG-101 (Group 2, n = 10) under fluoroscopic guidance in injured IVDs. The remaining discs i.e. adjacent healthy IVDs (L2/3, L4/5 and L6/7) served as no treatment controls (NTCs) in both the groups. At the end of experiment (i.e.14 weeks post injection) the animals were humanely euthanized, and each lumbar vertebral motion segment was dissected aseptically.

### Magnetic Resonance Imaging (MRI)

MR Imaging (MRI) was performed using a 3 Tesla Verio MRI system (IMRIS, Minnetonka, MN), with dogs (n = 5) in prone position and feet - first orientation. A 24-element spine matrix radiofrequency (RF) coil was posterior to the animal, and a 4 -element small flexible RF coil was positioned anterior to the lumbar region. The RF coils were purchased via IMRIS Inc., Winnipeg, Manitoba, Canada. The lumbar discs were first visualized using a stack of at least 3 sagittal-oriented 2D fat-suppressed T2-weighted images (echo time 83 ms, repetition time 4000 ms, 320 × 240 matrix over a 15 × 15 cm field-of-view providing 0.6 × 0.5 mm in-plane resolution, 3 mm slice thickness, 230 Hz/pixel readout bandwidth, 2 averages, 4 min 10 sec acquisition time).

### Disc Height Analysis in Chondrodystrophic (CD) Canines

Disc height analysis in CD canines (n = 16) was performed using fluoroscopic images obtained using Sedecal Dragon SPSLW digital X-ray system at baseline and the endpoint (i.e. 14 weeks post-intradiscal injection). Acquired radiographs were saved as DICOM images and using MicroDicom software (microdicom.com) and disc height was calculated from the superior to the inferior vertebral endplate. For details, see Supplementary Data.

### Histology, Immunohistochemistry (IHC) and Gene Expression Analysis

Hematoxylin and eosin (H&E) and Safranin-O staining was performed on paraffin-embedded sections (5 µm) to assess general morphology and proteoglycan content^18^. Histological grading of IVD-NP (injury followed by treatment) was carried out based on IVD – NP morphology, cellularity and Safranin O staining intensity in paraffin embedded sections following criterion described for rat^21,22^ and canine IVDs^23^ (Supplementary Tables 1–3). The histological scoring was done by 3 observers (ME, AM and HG), and scores were recorded independently. In the case of inter-observer variability of scores, all 3 observers reviewed scores and arrived at a final consensus score. Immunohistochemistry for ECM proteins (Aggrecan, Collagen 2A1), notochordal cell marker (Brachyury), stem cell marker (Oct4), pro-inflammatory cytokine (IL-1β and IL-6), ECM degrading enzyme (Matrix metalloproteinases-13, MMP-13) and inflammation association and pain related enzymes (Cyclooxygenase-2, Cox-2 and Prostaglandin E2, PGE2) was performed using the Vectastain Rabbit kit with Diaminobenzidine (DAB) as the chromogen^18^. Immunohistochemistry sections were analyzed semi-quantitatively using total of scores obtained based on % positivity and staining intensity for respective proteins^24^ (Supplementary Table 4). We performed quantitative Real Time - Polymerase Chain Reaction (qRT - PCR) to determine the effect of treatment on healthy ECM genes (Aggrecan, Collagen 2A1, hyaluronan and proteoglycan link protein 1, HAPLN1), inflammation and pain associated cytokines (IL-6 and IL-8) using species and gene specific primers with SYBR Green reagent. For details, see Supplementary Data.

### Biomechanical Analysis

We evaluated PBS (n = 4) or NTG-101 (n = 6) injected canine IVDs (L5/6) and adjacent lumbar spine segments, L6/7 serving as healthy no-treatment controls (n = 10). Custom software (LabVIEW, National Instruments) was used to test the specimens using an unconstrained force-control system. Force and moment targets were achieved by adjusting the velocity on each axis in proportion to force or moment errors while limiting the maximum velocity of the system. Flexion, extension, lateral bending and axial rotation were performed with three cycles each. Testing velocity was 0.1 degree per second with a maximal moment of 2.0 Nm. The resulting angle vs. moment data were then determined^25^. For details, see Supplementary Data.

### Statistical Analysis

Histograms representing mean ± standard deviation (S.D.) were plotted for all outcomes reported in this study. Statistical analysis was performed using Student’s t-test (paired/unpaired), Analysis of variance (ANOVA), Wilcoxon Signed Rank test and Mann-Whitney U test using SPSS 16.0 software (Chicago, IL, USA). P-value ≤ 0.05 was defined as statistically significant for all tests.

## Results

### Evaluation of the Anabolic Effects of the Combination of rhCTGF (100 ng/ml) and rhTGF-β1 (10 ng/ml) Proteins on Human NP Cells

**Figure 1:**
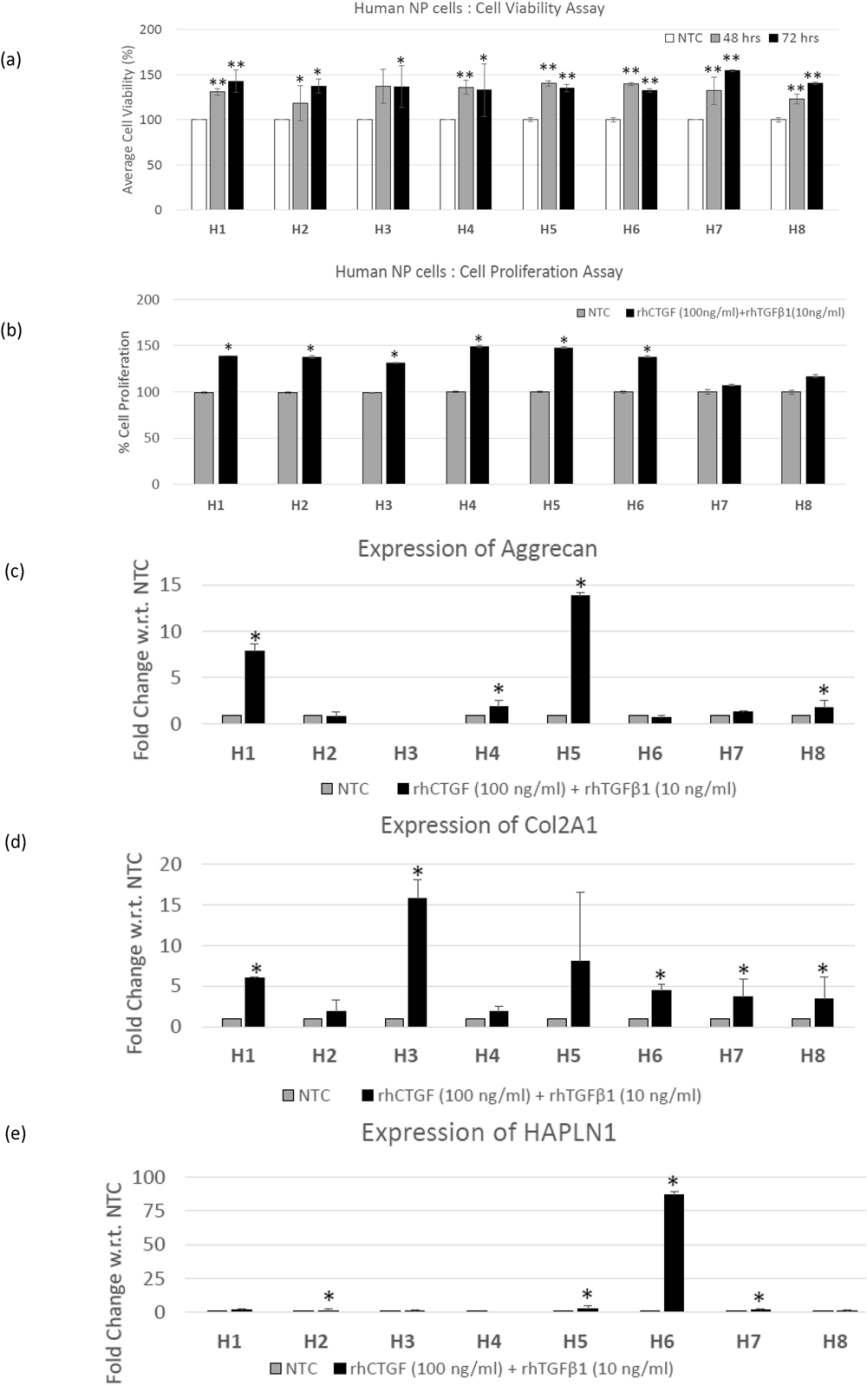
Pro-anabolic effects of combination treatment on human NP cells. (**a**) Panel shows histograms representing average cell viability (%) of human NP cells (H1- H8) treated with the combination of rhCTGF and rhTGF-β1 proteins for 48 hrs and 72 hrs in comparison to No treatment controls (NTC, shown by single bar as a representative for % cell viability 48 hrs and 72 hrs) as determined using cell viability assays (*p ≤ 0.05, **p < 0.005). (**b**) Panel shows histograms representing average cell proliferation (%) of human NP cells (H1 - H8) treated with the combination of rhCTGF and rhTGF-β1 proteins for 72 hrs as compared to No treatment controls (NTC) determined using BrdU enzyme linked immunosorbent assay (ELISA) assay (*p ≤ 0.05). Total RNA was extracted human NP cells (H1- H8) treated with combination treatment or left untreated in growth medium for 24 hrs to determine the effect of treatment on expression of healthy ECM genes. qRT-PCR analysis showing mRNA expression levels of (**c**) aggrecan, (**d**) Col2A1 and (**e**) HAPLN1 in human NP cells (H1- H8) treated with the combination of rhCTGF and rhTGF-β1 proteins for 24 hrs in comparison to their respective No treatment controls (NTC, *p ≤ 0.05).

Treatment with a combination of rhCTGF (100 ng/ml) and rhTGF-β1 (10 ng/ml) significantly increased the viability of human NP cells derived from all the donors within 48 hrs – 72 hrs as compared to their respective no treatment controls for specific time points (Fig. 1a, Supplementary Figure 1a). The increase in cell viability of human NP cells was further supported by increased cell proliferation in these cells on treatment with a combination of rhCTGF and rhTGF-β1 over 72 hrs as shown by increased bromodeoxyuridine (BrdU) incorporation (Fig. 1b, Supplementary Figure 1b). In addition, treatment with this combination induced a significant increase in expression of healthy ECM proteins including aggrecan, Col2A1 and HAPLN1 in human degenerative disc NP cells within 24 hrs (Fig. 1c–e, Supplementary Figure 1c). We observed a significant increase in aggrecan mRNA levels in human NP cells in at least 50% of the donors (H1, H4, H5 and H8, Fig. 1c), while increased Col2A1 expression was observed in all the donors (Fig. 1d). Treatment with combination of rhCTGF (100 ng/ml) and rhTGF-β1 (10 ng/ml) proteins increased HAPLN1 mRNA levels in human degenerative disc NP cells derived from 4 of the 8 (H2, H5, H6 and H7) donor patients (Fig. 1e).

### Evaluation of Anti-Catabolic Effects of Combination Treatment [rhCTGF (100 ng/ml) + rhTGF-β1 (10 ng/ml)] on Human NP Cells

**Figure 2:**
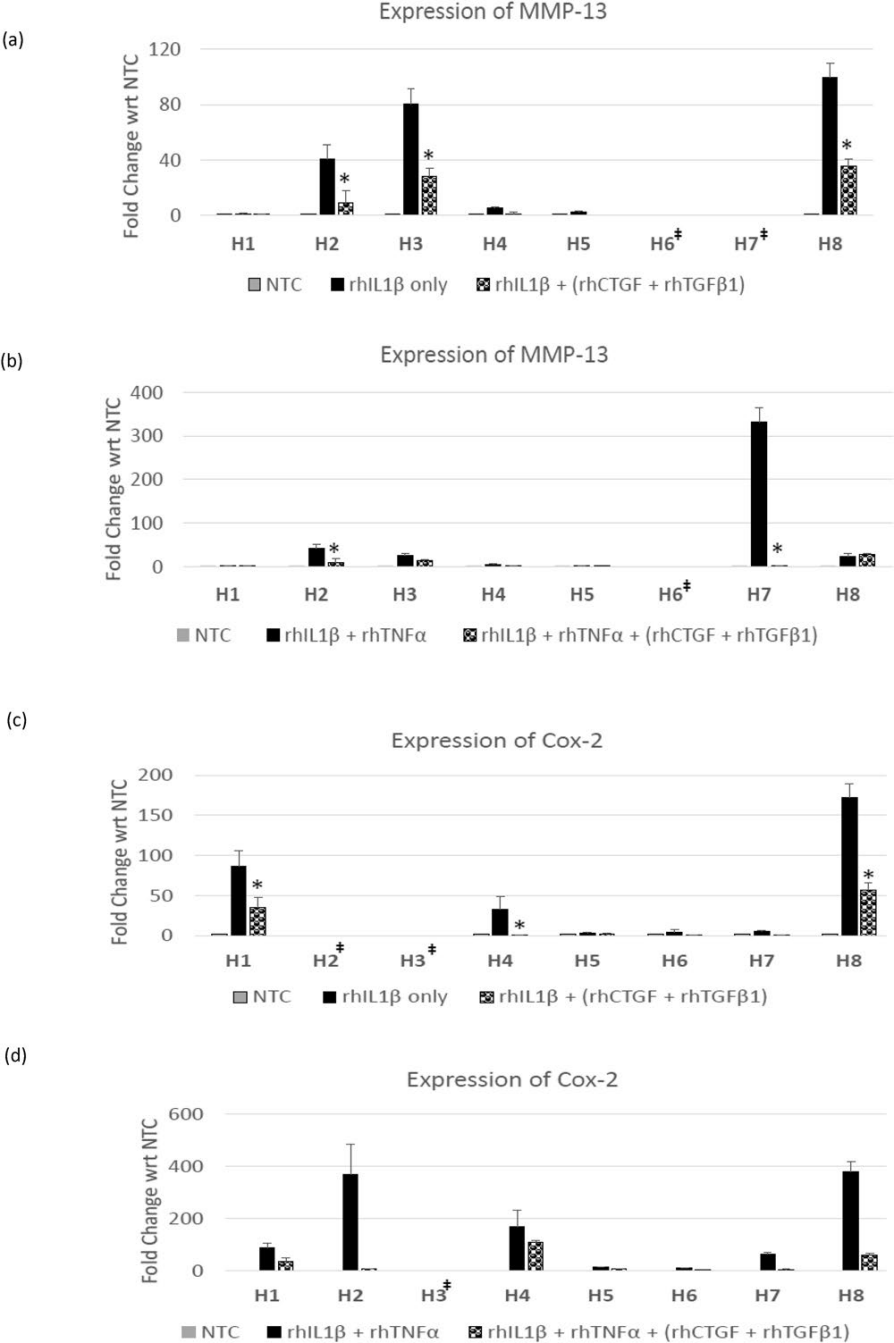
Anti-inflammatory effects of combination treatment on human NP cells. Panel shows histograms representing expression of (**a**,**b**) MMP-13 or (**c**,**d**) Cox-2 mRNA levels in human degenerative IVD - NP cells (H1–H8) treated with (**a** and **c**) rhIL-1β alone; (**b** and **d**) rhIL-1β + rhTNFα only or in the presence of a combination of rhCTGF (100 ng/ml) + rhTGFβ1 (10 ng/ml) as revealed by qRT-PCR analysis. p-values for combination of rhIL-1β/rhIL-1β + rhTNFα + rhCTGF + rhTGF-β1 are with respect to rhIL-1β alone or rhIL-1β + rhTNFα alone respectively (*p ≤ 0.05). ^‡^These samples did not show expression of MMP-13 or Cox-2 on treatment with rhIL-1β alone or combination of rhIL-1β + rhTNFα.

Human NP cells derived from patients (n = 8, H1- H8) undergoing spinal surgery were treated with pro-inflammatory cytokines, rhIL-1β (10 ng/ml) alone or in combination with rhTNFα (50 ng/ml) for 24 hrs. Treatment with rhIL-1β alone or a combination of rhIL-1β + rhTNFα induced expression of MMP-13 and Cox-2 in human NP cells (Fig. 2a–d). Notably, addition of a combination of rhCTGF (100 ng/ml) and rhTGF-β1 (10 ng/ml) proteins in the culture medium, reduced expression of MMP-13 and Cox-2 mRNA in degenerative disc NP cells treated with rhIL-1β alone or in combination with rhTNFα within 24 hrs (Fig. 2a–d).

### Histological Evaluation of Rat-Tail IVD - NP Tissue Sections (Healthy/PBS/Excipient Solution (ES)/NTG-101)

**Figure 3:**
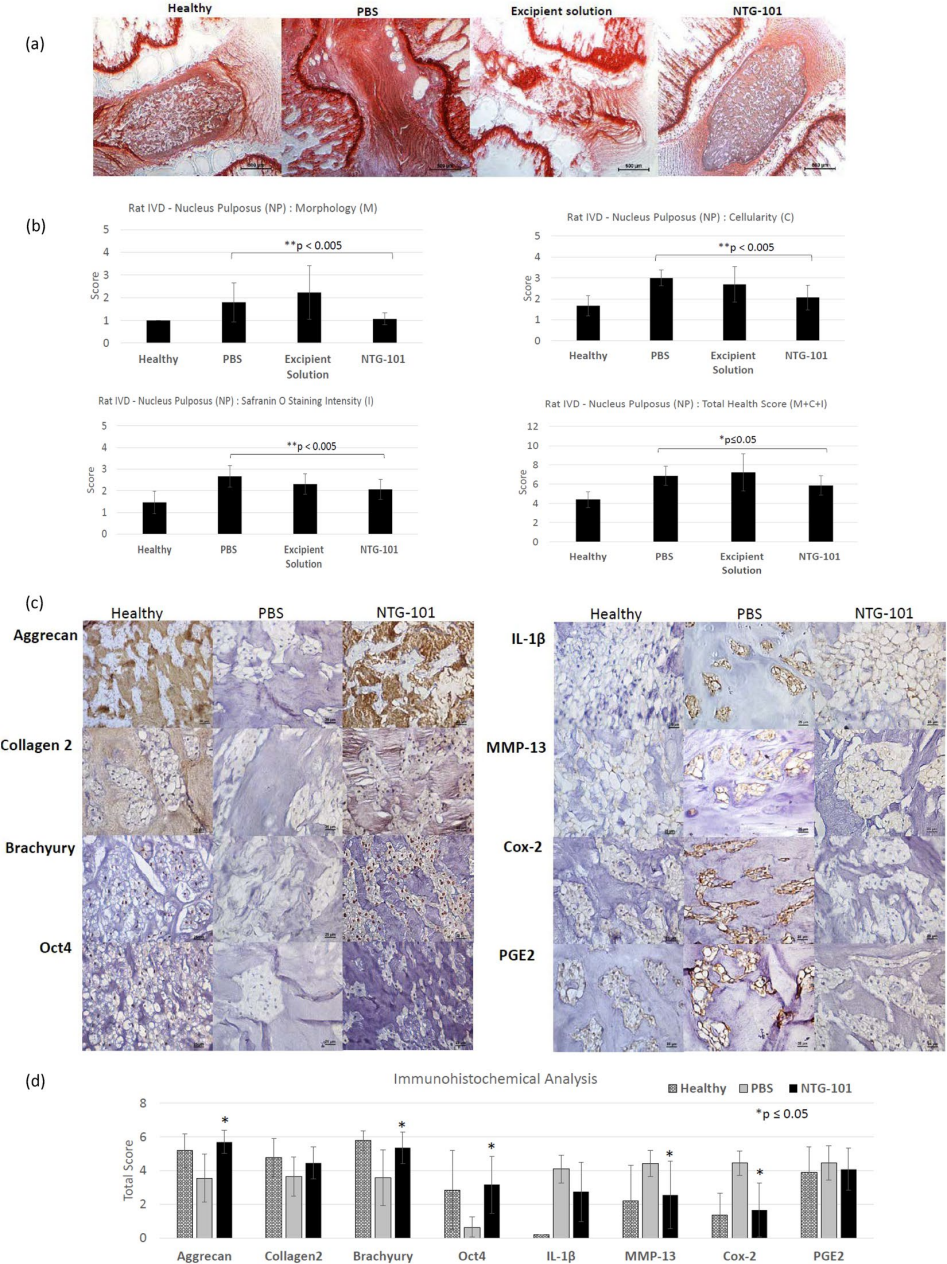
Effect of intra-discal injection of NTG-101 in rodent needle puncture disc injury model. (**a**) Representative images showing Safranin-O stained rodent IVD tissue sections (uninjured, healthy age-matched control), injured IVDs injected with either PBS (1X, pH = 7.2), excipient solution or NTG-101 (Scale bar: 500 µm). Panel (b) shows the histogram analysis showing mean score ± S.D. of the morphology (M), cellularity (C), Safranin O staining intensity (I) and total health score (M + C + I). ***p-value for NTG-101 injected group of animals is with respect to the group of injured IVDs injected with PBS only. Panel (c) represents images of immunohistochemistry for Aggrecan, Col2A1, Brachyury, Oct4, IL-1β, MMP-13, Cox-2 and PGE2 in age-matched, uninjured, healthy rat tail IVDs as well as injured IVDs injected with PBS (1X) or NTG-101 (Scale bar: 25 µm). Panel (d) represent histograms showing average values of IHC total score obtained for aggrecan, Col2A1, Brachyury, Oct4, IL1-β, MMP-13, Cox-2 and PGE2. Each histogram shows mean ± SD, *p ≤ 0.05.

We evaluated the scores for morphology (M), cellularity (C) and Safranin-O staining intensity (I) in tissue sections (n = 15, done in duplicates per group) obtained from rodent injured IVD – NPs that received an intra-discal injection of PBS, excipient solution or NTG-101 and untreated, non-injured age-matched healthy control IVD-NPs. The IVD-NPs that received an intra-discal injection of vehicle or excipient solution, showed distinguished features of disc degeneration with small, low cellularity or acellular IVD - NP with metaplasia of annulus fibrosus showing intense Safranin O staining. We observed the significant loss of cellularity and a strong Safranin O staining in PBS injected IVDs in comparison to healthy controls demonstrating the development of a degenerative phenotype (Fig. 3a and b). However, rat injured IVDs injected with NTG-101 showed no significant difference in scores for morphology, cellularity, Safranin-O staining intensity and total score for histology as compared to healthy controls (Fig. 3a and b).

### Immunohistochemical Analysis of Rat IVD - NP Tissue Sections (Healthy/PBS/NTG-101)

Immunohistochemical analysis was performed on serial tissue sections used for Safranin O staining to determine the expression of important proteins in rat-tail IVDs that received an intra-discal injection of NTG-101, PBS and healthy age-matched (22-week-old) rat IVD - NP tissues. We observed moderate/intense aggrecan and Col2A1 immunostaining in ECM and nuclear Brachyury, Oct4 expression in notochordal cell rich IVD-NPs injected with NTG-101, similar to healthy controls IVDs. However, injured IVDs injected with vehicle showed loss or reduced levels of these proteins (Fig. 3c and d). In fact, we observed a significant increase in inflammation and associated proteins including IL-1β, MMP-13 and Cox-2 levels in rat-tail IVDs injected with PBS (1X, pH = 7.2) following needle puncture injury (Fig. 3c and d). However, NTG-101 injected IVD - NPs demonstrated no or low levels of IL-1β, MMP-13 and Cox-2 suggesting that NTG-101 suppresses inflammation within IVD - NPs following injury (Fig. 3c and d). No significant alterations in PGE2 levels were observed 10 weeks post-injection of either PBS or NTG-101 in rat tail IVD - NP tissue sections (Fig. 3c and d).

### MRI Assessment of CD-Canine IVDs

**Figure 4:**
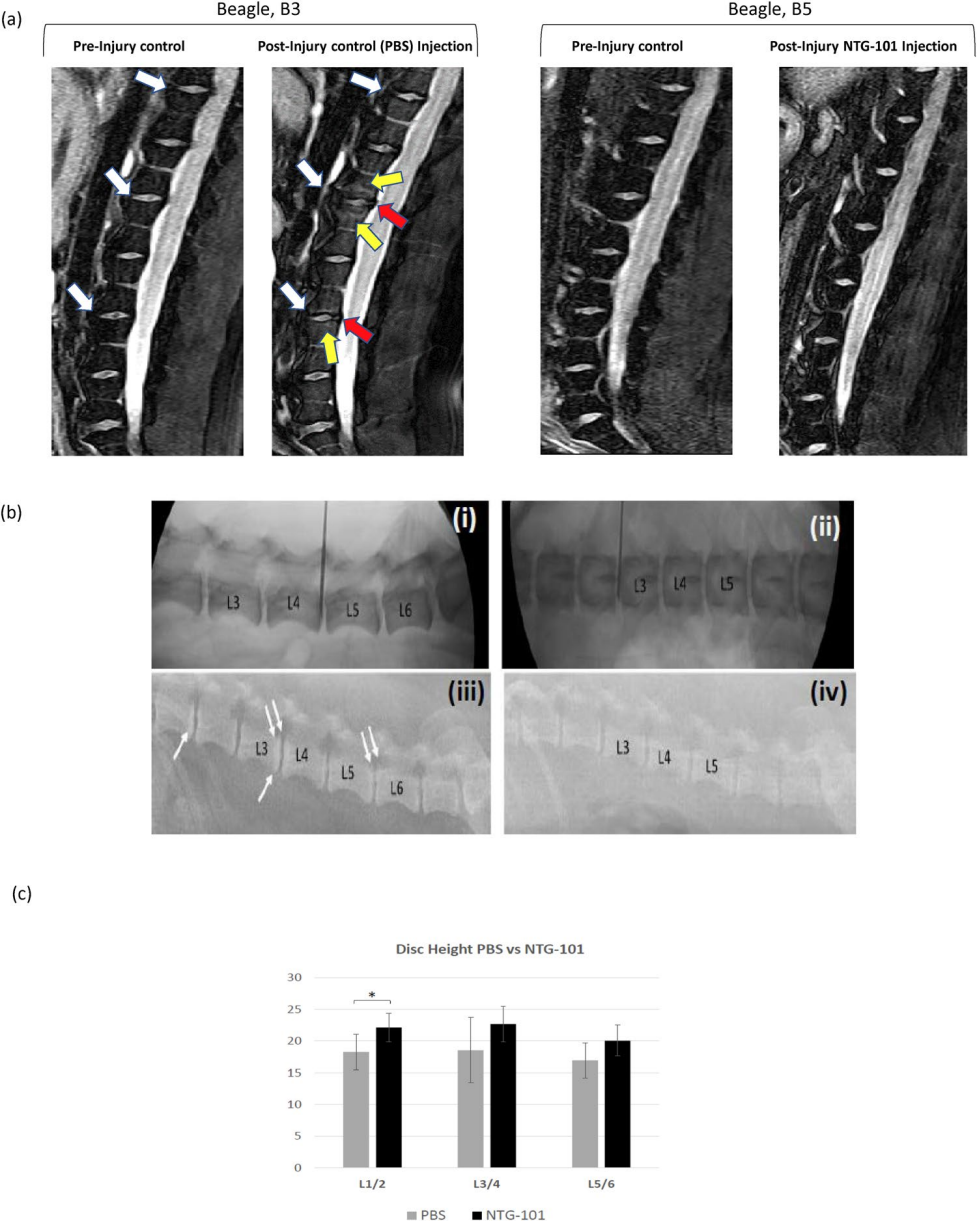
(**a**) Representative image showing 3T MRI of CD-canine (B3 and B5) lumbar spine with well-hydrated discs prior to injury (baseline MRI). In this image, white arrows show the targeted IVDs for needle puncture injury. Next image shows 3T MRI of CD-canine (B3) lumbar spine 18 weeks post-injury (i.e. 14 weeks post-treatment). The yellow arrows depict robust “modic” changes within bone marrow abutting the vertebral endplate that were completely absent prior to injury. Osteophytes were observed at spinal levels (L3/4 and L5/6) 18 weeks post-injury indicating the development of DDD in vehicle controls (red arrows). While CD-canine (B5) that received an intra-discal injection of NTG-101 represented MRI scans showing no significant difference from baseline images i.e. prior to injury. (**b**) Representative radiographic images of beagle, B3 (PBS injected) and beagle, B5 (NTG-101 injected) at baseline (i and ii) and 14 weeks post injections (iii and iv). At baseline, there is no demonstrable DDD between animals that received PBS or NTG-101 (i and iii). However, at the endpoint there is evidence of DDD at L1/2, L3/4 and L5/6 in the B3 beagle (loss of disc height, endplate sclerosis) (iii) whereas in (iv) the B5 beagle shows no detectable DDD. White arrows depict sub-chondral sclerosis and double white arrows depict the development of marginal osteophytes at the vertebral endplates. (Note, needle visible in a and c was for placement purposes only and does not represent injection or needle injury). (c) Panel represents histograms showing change in mean values of the disc height at respective spinal levels (L1/2, L3/4 and L5/6) injected with NTG-101 or PBS (1X, pH = 7.2) where the L1/2 level shows a significant preservation of disc height in discs injected with NTG-101 (*p ≤ 0.05).

Among the CD-canines (n = 2) injected with PBS (1X, pH = 7.2), animal (B3) developed robust degenerative changes at the L3/4 and L5/6-disc spaces. These degenerative changes included extensive type 1 modic changes affecting L3/4 and to a lesser extent L5/6 with a loss in disc height and the development of marginal osteophytes at the posterior superior and inferior aspect of the vertebral body (Fig. 4a). It is noteworthy that the lumbar spine of this canine (B3) was normal at baseline and these phenotypic changes representing DDD occurred after disruptive needle puncture injur y only (Fig. 4a). Among the CD-canines (n = 3) that received an intra-discal injection of NTG-101, post-injury, no evidence of DDD was observed 14 weeks post injection and all IVDs appeared identical to baseline (Fig. 4a, Supplementary Figure 2). No change was observed in adjacent, uninjured IVDs irrespective of the intervention given to animals (Fig. 4a, Supplementary Figure 2).

### Disc Height of CD-Canine IVDs

At baseline, no statistically significant difference was observed in the disc heights for all animals (n = 16, Supplementary Figure 3). However, IVDs injected with PBS (1X, pH = 7.2) post injury, demonstrated clear radiographic evidence of DDD with the development of marginal osteophytes at the vertebral endplate/vertebral interface (in a representative image of specimen B3) at the L3/4 and L5/6-disc spaces (Fig. 4b). There was clear evidence at the L1/2 spinal level that discs injected with PBS (1X, pH = 7.2) showed a significant reduction in disc height as compared to NTG-101 injected IVDs (p ≤ 0.05, Fig. 4c).

### Morphological and Histological Evaluation of CD-Canine IVD – NPs

**Figure 5:**
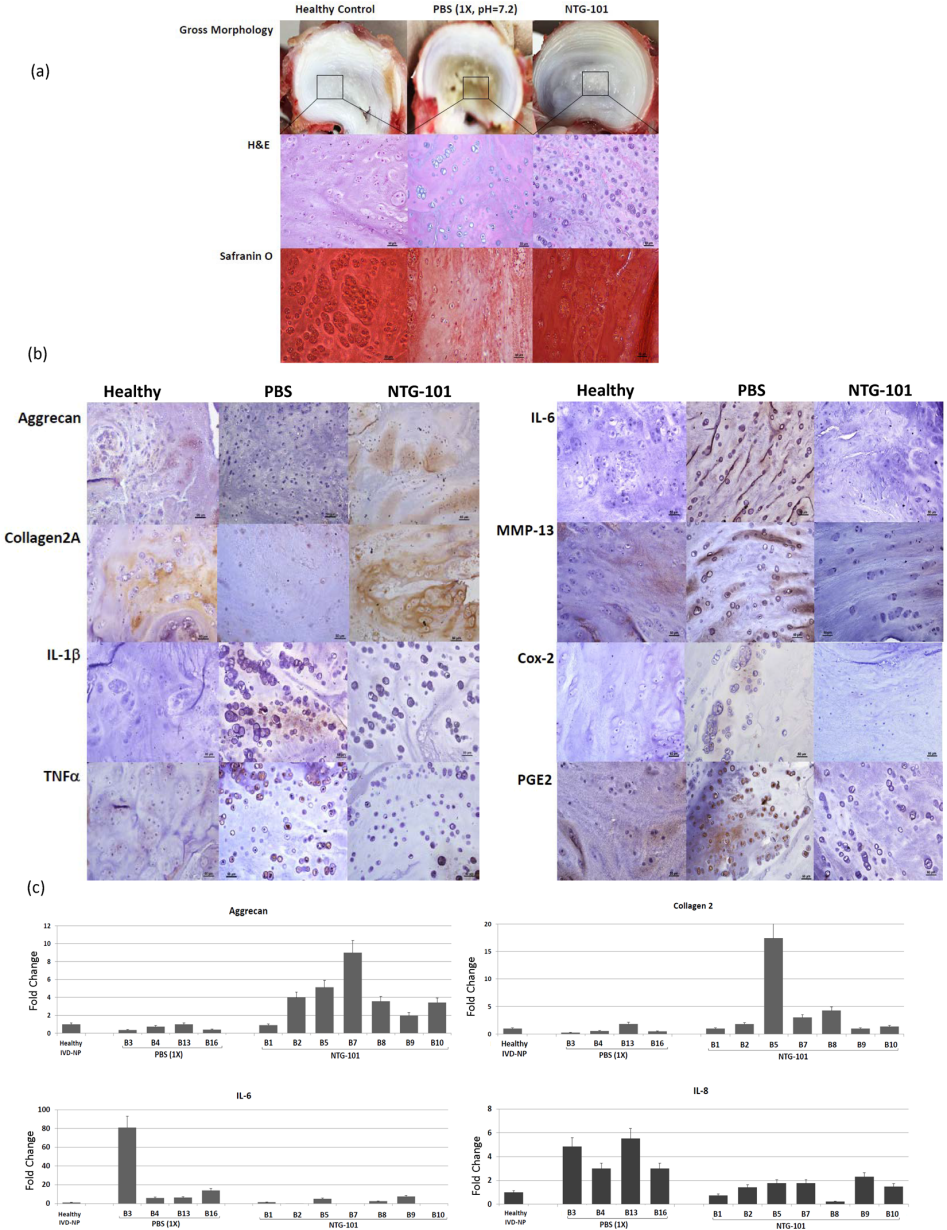
(**a**) Representative images showing gross morphology of the CD-canine IVDs at spinal level (L3/4) that received an intra-discal injection of PBS or NTG-101 and adjacent IVD at spinal level (L4/5) representing uninjured IVD. Hematoxylin and Eosin (H&E) stained sections revealed cellularity while Safranin O staining represented proteoglycan content. Healthy (no treatment) controls reveal normal IVD - NP morphology with good cellularity and intact ECM. IVDs injected with PBS (1X, pH = 7.2) displayed markedly disturbed cellularity and ECM whereas NTG-101 injected IVDs were comparable to adjacent, uninjured IVD-NPs (Scale bar: 50 µm for H&E, Safranin O stained sections). (**b**) Immunohistochemical analysis of aggrecan, collagen type 2A1, IL-1β, TNFα, IL-6, MMP-13, Cox-2 and PGE2 levels in uninjured controls, PBS and NTG-101 injected IVD – NPs (Scale bar: 50 µm). (c) Panels shows histograms representing relative fold change in mRNA levels of healthy ECM genes (aggrecan, Col2A1) and inflammation, pain associated genes (IL-6 and IL-8) expression in CD - canine IVD - NP tissues that received a single intra-discal injection of NTG-101 or PBS (1X, pH = 7.2) with respect to adjacent healthy, uninjured IVD-NPs obtained from the same animal. All gene expression fold changes for the injected IVD- NPs were normalized to expression levels in the individual adjacent, healthy canine disc using HPRT used as housekeeping gene. In Fig. 5, data from adjacent, healthy canine IVD-NPs is shown as a representative single bar.

We evaluated the gross morphology and histology (cellularity and proteoglycan content) of canine IVDs injected with either NTG-101 or PBS (1X, pH = 7.2) following needle puncture injury at spinal level L3/4 and compared it to adjacent uninjured, healthy IVD – NPs (spinal level, L4/5). Gross morphology of the IVD – NPs revealed reduced size and fibro-cartilaginous appearance of the NP in 4 of the 6 beagle IVDs that received an intra-discal injection of PBS (1X, pH = 7.2) as compared to adjacent, uninjured IVDs (Fig. 5a). Among the CD-canine IVDs that received an intra-discal injection of NTG-101, gross morphology was like adjacent uninjured, control IVDs (Fig. 5a). Histological evaluation demonstrated moderate cellularity with clusters of chondrocyte like-cells (CLCs) in uninjured, control or NTG-101 injected IVD – NPs as revealed by H&E and Safranin O staining (Fig. 5a, Supplementary Figure 4). In contrast, the IVD - NPs injected with PBS (1X, pH = 7.2) displayed a lack of chondrocyte like cells (CLC) - clusters in H&E and decrease in Safranin O staining (Fig. 5a, Supplementary Figure 4).

### Immunohistochemical Analysis of Canine IVD – NPs

Our IHC analysis revealed low or no detectable levels of ECM proteins including aggrecan and Col2A1 in CD-canine injured IVDs injected with PBS (1X, pH = 7.2). On the other hand, NTG-101 injected IVDs demonstrated moderate or intense aggrecan and Col2A1 immunostaining within the ECM surrounding NP cells, similar to that observed in uninjured, control IVDs demonstrating the pro-anabolic effects of NTG-101 (Fig. 5b). However, PBS (1 × , pH = 7.2) injected IVD - NP tissues demonstrated a moderate to strong immunostaining for pro-inflammatory cytokines (IL-1β, TNFα and IL-6) and inflammation induced MMP-13, Cox-2 and PGE2 levels (Fig. 5b). Whereas NTG-101 injected IVD - NPs revealed cellular clusters that were devoid of IL-1β, TNFα, IL-6, MMP-13, Cox-2 and PGE2 immunostaining like control IVD-NP tissues (Fig. 5b) clearly supporting the anti-inflammatory role of NTG-101 (Fig. 5b).

### Gene Expression Analysis of CD-Canine IVD – NPs

Fourteen weeks post - treatment, total RNA was extracted from CD-canine IVDs that received an intra-discal injection of PBS (1X, pH = 7.2) or NTG-101 at spinal levels (L1/2) and from adjacent, uninjured control IVDs at spinal level (L2/3). Gene expression of aggrecan, Col2A1, IL-6 and IL-8 was determined in injured IVD – NP tissues injected with NTG-101, PBS (1X, pH = 7.2) and control IVDs using qPCR with canine gene specific primers. Quantitative real time PCR results revealed an increase in aggrecan and Col2A1 gene expression in NTG-101 injected IVD-NPs in comparison to PBS – injected IVD-NP tissues and significant reduction in gene expression levels of both the pro-inflammatory cytokines, IL-6 and IL-8 as compared to PBS-injected IVD – NPs (Fig. 5c, Supplementary Figure 4).

### Biomechanical Analysis of Canine – IVDs

**Figure 6:**
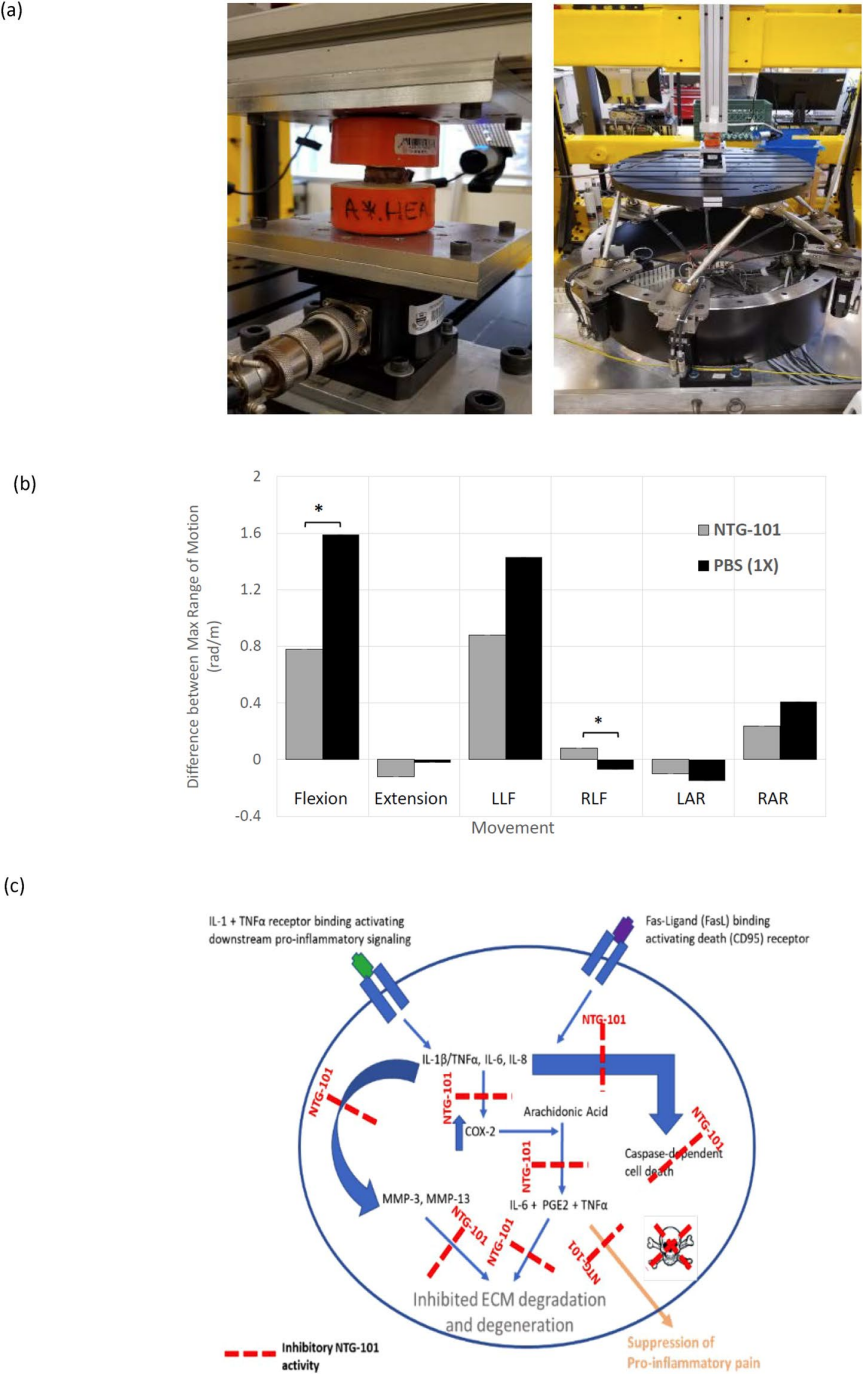
Close-up of vertebral segment within testing frame. (**a**) Full view of hexapod (6-axis load cell) testing robot. (**b**) Biomechanical assessment of degenerative L5/6 IVD motion segments injected with NTG-101 or PBS. The specimens’ properties (under flexion and right lateral flexion loading) of the NTG-101 injected discs are statistically significantly preserved compared to those injected with PBS (*p ≤ 0.05). (**c**) Model representing mechanism of action (MoA) of NTG-101 showing its anti-inflammatory, anti-catabolic and pro-anabolic effects in a degenerative IVD-NP. Treatment with NTG-101 suppresses inflammation induced expression of matrix degrading enzymes (MMP-3, MMP-13), cell death and pain associated molecules (Cox-2, PGE2).

We performed repeated measures of the maximum rotational angle (a measure of how much the sample changes over time which is a measure of the viscoelastic properties of the sample) achieved using the NTG-101 or PBS-injected IVDs (L5/6) and compared these changes to the adjacent, uninjured IVD (L6/7) that served as a no treatment control (Fig. 6a). The absolute and normalized values (range, mean ± SD) for flexion, extension, left lateral flexion (LLF), right lateral flexion (RLF), left axial rotation (LAR) and right axial rotation (RAR) for CD-canine IVDs injected with either NTG-101 or PBS (1X) (Tables 1, 2 and Fig. 6b). For normalization, absolute values were normalized with respect to IVD height for each specimen. We observed significant differences in normalized values for flexion and right lateral flexion (RLF) in the PBS-injected IVDs in comparison to the IVDs injected with NTG-101 (p ≤ 0.05, Tables 1, 2 and Fig. 6b).

**Table 1:** Absolute values of flexion, extension, lateral bending and axial rotation in CD-canine treated IVD segments.

Biomechanical Characteristics	NTG-101 Range (Mean ± SD) (rad)	PBS (1X) Range (Mean ± SD) (rad)
Flexion	0.01–0.02 (0.02 ± 0.00)	0.02–0.03 (0.02 ± 0.01)
Extension	−0.01–0.00 (0.00 ± 0.01)	−0.01–0.00 (0.00 ± 0.00)
Lateral bending		
Left	0.01–0.03 (0.02 ± 0.01)	0.01–0.03 (0.02 ± 0.01)
Right	0.00–0.00 (0.00 ± 0.01)	0.00–0.00 (0.00 ± 0.01)
Axial Rotation		
Left	0.00–0.00 (0.00 ± 0.00)	0.00–0.00 (0.00 ± 0.00)
Right	0.00–0.02 (0.01 ± 0.00)	0.00–0.01 (0.01 ± 0.00)

**Table 2:** Normalized values of flexion, extension, lateral bending and axial rotation in CD-canine treated IVD segments.

Biomechanical Characteristics	NTG-101 Range (Mean ± SD) (rad)	PBS (1X) Range (Mean ± SD) (rad)
Flexion	0.50–0.88 (0.78 ± 0.19)	1.21–1.99 (1.59 ± 0.39)
Extension	−0.49–0.22 (−0.12 ± 0.3)	−0.061–0.01 (−0.02 ± 0.04)
Lateral bending		
Left	0.35–1.14 (0.88 ± 0.36)	0.81–1.91 (1.43 ± 0.56)
Right	0.00–0.24 (0.08 ± 0.11)	−0.12–−0.04 (−0.07 ± 0.04)
Axial Rotation		
Left	−0.14–−0.05 (−0.01 ± 0.04)	−0.20–−0.07 (−0.15 ± 0.07)
Right	0.12–0.39 (0.24 ± 0.11)	0.21–0.53 (0.41 ± 0.17)

## Discussion

Both CTGF and TGF-β1 are vital elements during embryological development of the IVD and cartilage, as well as the post-natal stage^26,27^. Further, CTGF and TGF-β1 are vital to the homeostatic regulation of the IVD-NP^18^. Ageing, inflammation and other degenerative conditions along with lack of CTGF and TGF-β1 lead to dysregulated signaling pathways leading to progression of DDD^18,28^. However, the notochordal cell rich IVD-NP in NCD-canines that secrete CTGF and TGF-β1 resist the development and progression of DDD^18^. We have previously demonstrated that a single injection of NCCM into the injured/degenerative rat-tail IVD-NPs conferred a robust anti-degenerative and pro-anabolic effect that was recapitulated by a single injection of a combination of rhCTGF + rhTGF-β1 proteins^18^. In the current study, we evaluated the effect of this combination treatment on cell viability, proliferation, gene expression of healthy ECM proteins (aggrecan, col2A1, HAPLN1) and inflammation induced expression of MMP-13 and Cox-2 in human degenerative IVD-NP cells. Our results demonstrated pro-anabolic and anti-inflammatory effects of the combination of rhCTGF and rhTGF-β1 in human NP cells. Treatment with the combination of these growth factors induced cell viability and proliferation as well as exhibited robust ECM protein synthesis while suppressing inflammation. Further, our *in vivo* results revealed no significant difference in total histological scores for rat-tail injured IVD-NPs that received an intra-discal injection of higher doses of rhCTGF (200 ng/ml) or rhTGF-β1 (20 ng/ml) in comparison to rhCTGF (100 ng/ml) or rhTGF-β1 (10 ng/ml) respectively (data not shown). Considering the therapeutic potential of this combination both in *in vitro* (human and rodent) and *in vivo* rodent disc injury model, we formulated NTG-101 containing rhCTGF (100 ng/ml) and rhTGF-β1 (10 ng/ml) in an excipient solution and tested its efficacy in two independent *in vivo* models of DDD following needle puncture injury (i.e. rodent and CD-canines). The CD-canine has been validated as an appropriate model of human disease by our group and others further supporting the use of this large animal model to investigate the progression of DDD and evaluate the biological therapy^25,29^.

Interestingly, we found that a single injection of NTG-101 conferred a robust pro-anabolic and anti-catabolic effects in the CD-canine model indicating that our intervention can restore homeostatic regulation within degenerative IVD-NPs. On the other hand, we found that the injured canine IVDs that were injected with vehicle degenerated in a fashion akin to the human IVDs. These signs of degeneration include modic changes, and loss of disc height as well as other molecular, histological and biomechanical changes consistent with DDD^30,31^. Among animals that received an intradiscal injection of the vehicle post-injury, we observed loss of disc height, sub-chondral sclerosis and formation of marginal osteophytes as revealed by radiographic analysis. These IVD-NPs also showed low cellularity, decreased expression of aggrecan and Col2A1 while over expressing the pro-inflammatory cytokines (IL-1β, IL-6, IL-8, TNFα) suggesting the development of an inflammatory microenvironment in the IVD-NP. These observations are also supported by prior studies emphasizing the possible role of pro-inflammatory cytokines and pain of disc origin^32^. Interestingly, Burke *et al*.^33^, reported that disc tissues obtained from back pain patients undergoing surgery displayed significantly elevated levels of IL-6, IL-8, and PGE2 than did patients with sciatica or as compared to normal. In contrast, the IVDs of the NTG-101 injected animals demonstrated no significant changes on MRI imaging and radiographs as compared to the adjacent, healthy IVDs at the endpoint. In addition, these IVD-NPs maintained a healthy disc phenotype showing cellular IVD-NPs with expression of aggrecan, Col2A1 like adjacent healthy IVDs. We also observed reduced expression of inflammation and associated proteins (IL-1β, IL-6, IL-8, TNFα, Cox-2 and MMP-13) clearly suggesting the pro-anabolic and anti-inflammatory role of NTG-101 in degenerative IVD-NPs. These results strongly suggest that a single injection of NTG-101 into the degenerative disc can overcome the injury and pro-inflammatory effects conferred by needle puncture injury and induce repair. However, the degenerative disc derived human NP cells showed a heterogenous response on treatment with the combination of rhCTGF + rhTGF-β1 proteins within 24 hrs – 72 hrs in this study. These human IVD-NP primary cell cultures containing mixed populations of NP and likely AF and endplate - cells were derived from patients undergoing discectomy and pose a major challenge in *in vitro* studies^34^. Further, the lack of cellular and molecular markers for NP cells adds to this complexity^35^. Nevertheless, our study established the proof of principle and large-scale studies including safety and toxicity as well as efficacy of NTG-101 in patient derived NP - cells and clinical trials are underway to determine the clinical utility of NTG-101.

The progression of DDD is marked by the development of a progressively pro-inflammatory milieu within the NP leading to degradation of healthy ECM molecules including aggrecan and collagen type 2 (Fig. 6c). This results in loss of hydration, disc height and impaired biomechanical properties driven by inflammation demonstrating homeostatic dysregulation in the degenerative IVD-NP^31,36^. There are myriad events involved with these pathological changes but increased IL-1β and TNFα levels are fundamental to driving inflammation-related progressive degeneration^18,37^. We have previously demonstrated increased levels of IL-1β and TNFα in IVD-NPs following needle puncture injury in our rodent model of DDD^18^. The net result of sustained exposure to these pro-inflammatory cytokines within the degenerative IVD-NP results in degradation of the ECM, loss of viable cells and diminished structural and biomechanical properties of the IVD^32,38,39^. IL-1β is one of the major players involved with the progression of DDD due to its pro-inflammatory effects within the IVD-NP^33,37,38^. Over-expression followed by binding of IL-1β and TNFα results in increased transcription of pro-inflammatory cytokines including IL-1β, IL-6, IL-8 and TNFα, the ECM degrading enzymes MMP-3, MMP-13, A disintegrin and metalloproteinase with thrombospondin motifs −4/5 (ADAMTS-4/5) and inflammation/pain associated protein (Cox-2). Both IL-6 and IL-8 further promote inflammation resulting in a positive feedback loop that in turn increases expression of TNFα, MMP-3 and Cox-2^32^. The net result is the development of the pro-catabolic microenvironment within IVD-NP enriched in ECM degrading enzymes, inhibition of de novo ECM protein synthesis leading to impaired water binding within the NP, and the production of pain related molecules^1,32^.

Our data suggest that NTG-101 plays an important role in the suppression of inflammation and the restoration of ECM proteins (aggrecan, Col2A1, and HAPLN1) both *in vitro* and in *in vivo* (Fig. 6c). Among the components of NTG-101, both CTGF and TGF-β1 are known for their anti-inflammatory and cell growth promoting functions. The CTGF protein comprises insulin-like growth factor binding protein (IGFBP), von Willebrand factor C (VWC), thrombospondin (TSP) and Cystine knot (CT) domains that confer CTGF the ability to modulate growth factor signaling. The IGFBP domain is capable of binding to aggrecan and promote its production from chondrocytes^40^, while the VWC domain interferes bone morphogenetic protein-4 (BMP-4) dependent signaling but enhances the receptor binding to TGF-β1^41^. On the other hand, the TSP domain is important for adhesion, collagen deposition and the regulation of angiogenesis by CCN2/CTGF^42,43^. The CT domain that binds to heparan sulfate proteoglycans (HSPGs) and integrins (αVβ3 and α5β1) is critical for adhesion, mitogenic effects and extracellular matrix production^44–46^. In addition to its anabolic role, CTGF can also suppress IL-1β-induced mRNA levels of MMP-3 and ADAMTS5 through cell surface integrin receptors (αvβ3 and α5β1) in IVD-NP cells^28^. Of note, co-transduction of lentiviral vectors containing TGFβ3, CTGF and tissue inhibitor of metalloproteinsases-1 (TIMP1) genes induced significant expression of type II collagen and aggrecan, delaying IVD - degeneration in rabbit annular puncture model of DDD^47^. Further, Cai *et al*.^48^, reported the addition of rhTGF-β1 partially reversed the catabolic effects of conditioned medium obtained from degenerative human IVD-NP cells (dCM) but increased expression of tissue inhibitor of metalloproteinsases-1/2/3 (TIMP-1/2/3), aggrecan and Col2^48^. Recently, Bian *et al*.^49^, showed increased expression of CCN2/CTGF and aggrecan in response to TGF-β signaling activation, supporting our results. Another report by Tran *et al*.^28^, also suggested that TGF-β regulates CTGF expression via activation of transcription factor, activator protein 1 (AP1) representing an attempt at repair following injury in a murine model^28^. Within the context of these observations, it is noteworthy that both CTGF and TGF-β1 proteins are capable of activating Smad-2/3 dependent signaling to increase the transcription of proteoglycans such as aggrecan, collagen type 2 and HAPLN1^28,50,51^. CTGF can signal via complex interplay with Src and MAPKs to activate Smad-2/3 signaling^28^. Further using TGFβ-RII knock-out and lumbar spine instability mouse models, Bian *et al*.^49^, reported activation of TGF-β signaling following mechano-signaling transduction via integrins (αvβ6) plays an important role in maintaining homeostasis and cellular functions in IVD. However, this effect could be dose dependent as demonstrated by Bian *et al*.^52^, wherein authors showed supraphysiological levels of TGFβ can lead to end plate degeneration and be detrimental to IVD. In contrast to our findings, both CTGF and TGF-β1 have been reported to promote fibrosis in some connective tissue disorders^53^. However, within the hypoxic, ischemic, avascular and immune privileged IVD-NP, we did not observe development of fibrosis within the NP following injection of NTG-101. In fact, in our study this combination of proteins served to increase cell survival, synthesis of vital ECM molecules, and suppressed the pro-inflammatory/catabolic environment within the degenerative IVD-NPs.

Based on our results using *in vivo* models as reported here, it is tantalizing to hypothesize that an injection of NTG-101 may have similar effects on human degenerative IVD-NPs. In conclusion we provide a strong rationale for the use of NTG-101 as a novel, molecular therapy to treat DDD.

## Electronic supplementary material

Supplementary Data
