# Supplementary material for: NTG-101: A Novel Molecular Therapy that Halts the Progression of Degenerative Disc Disease
Source: Sci Rep. 2018 Nov 14;8:16809. doi: 10.1038/s41598-018-35011-4 (PMC6235869; doi:10.1038/s41598-018-35011-4)
Supplement: Supplementary file 1 — Supplementary Data [file 41598_2018_35011_MOESM1_ESM.pdf]

NTG-101: A Novel Molecular Therapy that Halts the Progression of Degenerative Disc Disease

Authors: Ajay Matta[1], Muhammad Zia Karim[1], Hoda Gerami[1], Peter Jun[2], Martha Funabashi[2], Greg Kawchuk[2], Alyssa Goldstein[3], Warren Foltz[3], Marshall Sussman[3], Bjorn C Eek[4], W. Mark Erwin[1,5]

Affiliations: [1]Notogen Inc., Toronto, Ontario, Canada.
[2]University of Alberta, Edmonton, Alberta, Canada.
[3]University Health Network, Toronto, Ontario, Canada.
[4]Private Practice, Irvine California
[5]Department of Surgery, University of Toronto, Toronto, Ontario, Canada.

Corresponding Author: *W. Mark Erwin DC, PhD
Email: mark.erwin@utoronto.ca; Tel: 1-647-243-5278

Short Title: Molecular therapy to treat degenerative disc disease

Key words: CTGF, TGF-β1, Degenerative Disc Disease, Nucleus pulposus, NTG-101, Inflammation

SUPPLEMENTARY DATA

Reagents, Cell Culture and Treatments for *in vitro* Analysis. Human recombinant Interleukin-1β (IL-1β), Tumor necrosis factor-alpha (TNFα), Connective tissue growth factor (CTGF) and Transforming growth factor beta 1 (TGF-β1) proteins were purchased from Peprotech Inc. (Quebec, Canada). We obtained rabbit polyclonal antibodies for aggrecan (ab216965), collagen 2 (ab34712), Cyclooxygenase-2 (Cox-2, ab15191), interleukin-1β (IL-1β, ab9722), interleukin-6 (IL-6, ab193853), MMP-13 (ab39012), Oct4 (ab18976), tumor necrosis factor alpha (TNFα, ab667), and prostaglandin E2 (PGE2, ab2318) were purchased from Abcam Inc. (CA, USA). Goat polyclonal brachyury antibody (sc-17743) was obtained from Santa Cruz Biotechnology Inc. (CA, USA).

Human degenerative disc nucleus pulposus tissues were obtained from patients (n = 8) undergoing discectomy or fusion surgery at Toronto Western Hospital, University Health Network (UHN), Toronto with all cases obtained in accordance with the guidelines approved by the Research Ethics Board, Toronto Western Hospital, UHN, Toronto. Nucleus pulposus (NP) tissue obtained at the time of spinal surgery was immediately transported to the laboratory and washed in phosphate buffered saline (PBS, 1X, pH = 7.4) followed by RBC lysis buffer (Invitrogen) was separated and enzymatically digested according to our established methods [15,16]. The next day, the cells were filtered with a 70 µm cell strainer (Falcon) and cultured within a hypoxic incubator (NuAire, MN, USA) in 3.5% O_2, 5% CO_2, in Advanced Dulbecco's modified Eagle's medium (ADMEM) supplemented with 8% fetal bovine serum (FBS), penicillin and streptomycin (100 U/mL) until passage (P2) as described earlier [17,18]. Thereafter the cells were either cultured in serum free ADMEM (no treatment controls) or treated with rhIL-1β (10 ng/ml), rhTNFα (50 ng/ml), or a combination of rhCTGF (100 ng/ml) + rhTGF-β1 (10 ng/ml) proteins for 24 hrs under hypoxic conditions to determine the effect of the treatment on cell viability, proliferation and ECM synthesis as described below.

Cell Viability, Cell Proliferation Assay and Gene Expression Analysis. Nucleus pulposus cells were plated in 96-well flat bottom plates to evaluate the effect of treatment with growth factors in cell viability and proliferation assays. Human degenerative disc NP cells were treated with a combination of rhCTGF (100 ng/ml) and rhTGFβ1 (10 ng/ml) for 48 hrs – 72 hrs to assess the effect of these growth factors on viability. Cell viability was determined using 3-(4,5-

dimethylthiazol-2-yl)-2,5-diphenyltetrazolium bromide (MTT, Sigma-Aldrich, USA) as described earlier [18]. We used a BrdU - ELISA (colorimetric) assay (Cat# ab126556, Abcam) to determine the effect of treatment with a combination of rhCTGF (100 ng/ml) and rhTGF-β1 (10 ng/ml) for 72 hrs on human degenerative disc NP cells following the manufacturer's instructions. Briefly, NP cells were treated with a combination of rhCTGF (100 ng/ml) and rhTGF-β1 (10 ng/ml) for 72 hrs following addition of BrdU reagent overnight (O/N) in each well. Incorporated BrdU in DNA of proliferating cells was determined using anti-BrdU antibody and quantified by ELISA as per the manufacturer's instructions. Quantitative real time - PCR analysis was performed to determine the effect of combination treatment (rhCTGF + rhTGF-β1) on ECM synthesis and anti-inflammatory effects on human degenerative IVD – NP cells. Total RNA was isolated from human degenerative disc NP cells (H1- H8) treated with rhIL-1β alone or in combination with rhTNFα, rhCTGF + rhTGF-β1 or untreated, no treatment controls (NTC) using Trizol and quantified using a Nanodrop spectrophotometer. Total RNA (~400ng) was reverse-transcribed using iScript (Biorad, CA) following the manufacturer's instructions for preparing cDNA. Effect of treatment on ECM genes including (Aggrecan, Col2A1, HAPLN1) and inflammation induced expression of (MMP-13, Cox-2) was evaluated using real time PCR performed on ABI 7900HT 384-well Fast block machine. For qRT-PCR, all data are expressed as mean ± standard deviation (S.D.). Fold changes of gene expression in IVD - NP tissues which received an intra-discal injection of NTG-101 or PBS (1X, pH = 7.2) was calculated with respect to adjacent healthy, uninjured IVD - NPs using $2^{-\Delta\Delta Ct}$ method. For gene expression analysis, p-values were determined for comparison of treated IVD - NPs with respect to their adjacent healthy controls.

Preparation of NTG-101. NTG-101 is a proprietary formulation containing a combination of rhCTGF (100 ng/ml) and rhTGF-β1 (10 ng/ml) proteins suspended in an excipient solution (ES). Stock solution of rhCTGF (100 µg/ml) was prepared in sterile water while rhTGF-β1 (10 µg/ml) was dissolved in 0.1 M citric acid. Both rhCTGF and rhTGF-β1 proteins were added in appropriate dilutions in excipient solution and pH of NTG-101 solution was adjusted to 7.2.

Immunohistochemistry. Following H&E and Safranin O staining, serial tissue sections were deparaffinized in xylene followed by hydration in gradient alcohol. Antigen retrieval was performed using microwave-based heat retrieval method. The slide sections were heated for 5 - 8 min. in Tris-EDTA buffer (1X, pH = 8.0) containing 0.1% - Tween or Citrate buffer (0.01 M, pH

= 6.0). They were then washed slides 3 times with Tris buffered saline (TBS, 1X, pH = 7.4) containing 0.025% Triton-X-100, followed by blocking with 10% species appropriate serum. The slides were then incubated with rabbit / goat polyclonal primary antibodies at appropriate dilutions for 1 hr at room temperature. The slides were then washed 3 times with Tris buffered saline (TBS-T, 1X, pH = 7.4, containing 0.025% Triton-X-100). The sections were incubated with hydrogen peroxide (0.3% v/v) for 15 minutes. Next, we used TBS-T to wash the slides 3 times followed by incubation with the respective secondary antibodies (rabbit / goat) at appropriate dilutions for 30 min. Protein expression was detected using the Vectastain ABC kit with diaminobenzidine (DAB) as chromogen. The sections were counterstained with Meyer's hematoxylin and mounted with DPX mountant. The bright field sections were evaluated semi-quantitatively for % positivity and staining intensity in IVD-NP by light microscopic examination using a Nikon bright field microscope. Immunohistochemistry total score for each protein of interest was calculated as sum of the scores obtained for % positivity and staining intensity in each tissue section as described earlier [24].

Disc Height Analysis in Chondrodystrophic (CD) Canines. At the beginning of the experiment (baseline) chondrodystrophic (CD) canines were anaesthetized under Veterinary supervision and placed on an operating table supported by a radiographically transparent plexiglass frame (Supplementary Figure 3a). Spinal radiographs were obtained for all animals at baseline obtained using a Phillips BV Pulsera C-arm Fluoroscopy unit. Next IVD - NP needle - puncture injuries were performed at selected levels (L1/2, L3/4 and L5/6) and one month later the same injured IVD - NPs were injected with either vehicle control (PBS, 1X, pH = 7.2) or NTG-101 and re-imaged 14 weeks later at the endpoint using the Sedecal Dragon SPSLW digital X-ray system. Needle puncture injuries of the IVD NPs were performed in the CD canine IVDs (n = 16) at the L1/2, L3/4 and L5/6 spinal levels under fluoroscopic guidance and Veterinary supervision. Acquired radiographs were saved as DICOM images and using MicroDicom software (microdicom.com), a minimum of 6 and up to 8 measurements of the disc space were obtained for each IVD. Calibration was performed prior to disc measurements using intrinsic MicroDicom software (Supplementary Figure 3b). Disc height was calculated from the superior to the inferior vertebral endplate. Using MS-Excel software, the mean IVD height was calculated for every disc (no treatment control and experimental discs). For disc height, p-values were calculated for IVDs that received an intra-

discal injection of NTG-101 or phosphate buffered saline (PBS, 1X, pH = 7.2) with respect to adjacent healthy.

Gene Expression Analysis in Canine IVD – NPs. Total RNA was isolated from CD healthy, uninjured IVD - NPs (at spinal level L2/3) and injured IVD – NPs injected with either NTG-101 or PBS (at spinal level L1/2) using Trizol and quantified using a Nanodrop spectrophotometer. Total RNA (~400ng) was reverse-transcribed using iSCript cDNA synthesis kit (Cat#1708890, Bio-Rad) following the manufacturer's instructions for preparing cDNA. We performed quantitative Real Time - Polymerase Chain Reaction (qRT - PCR) to determine the effect of treatment on healthy ECM genes (Aggrecan, Collagen 2, HAPLN1), inflammation and pain associated cytokines (Interleukin-6, IL-6 and Interleukin-8, IL-8) using species and gene specific primers with SYBR Green reagent. Hypoxanthine phosphoribosyltransferase (HPRT) was used as a house keeping gene for normalization of gene expression. qRT-PCR was performed on ABI 7900HT 384-well Fast block machine. Data analysis for qRT-PCR was carried out using calculation of ΔΔCt values. Fold changes were calculated with respect to no treatment controls (NTC) using $2^{-\Delta\Delta Ct}$ method as described earlier [16,18]. For gene expression analysis, p-values were determined for comparison of treated IVD - NPs with respect to their adjacent healthy controls.

Biomechanical Analysis. We evaluated PBS (n = 4) or NTG-101 (n = 6) injected canine IVDs (L5/6) and adjacent lumbar spine segments, L6/L7 serving as healthy no-treatment controls. All vertebral bodies were transected using a handsaw in the axial plane resulting in two motion segments bounded by hemivertebra ($L5^{1/2}$-disc-$L6^{1/2}$ and $L6^{1/2}$-disc-$L7^{1/2}$) creating 20 motion segments. To each hemivertebrae, a series of 6 screws (3 of #4 x ¾ inch, 3 of #6 x 1 inch) were inserted in a radial pattern with ~5/8-3/4 inch left to protrude. Each segment was then lowered into a plastic pot while controlling specimen alignment with a horizontal laser level (DeWalt Industrial Tool Co., Baltimore, MD), ensuring that the IVD was parallel to the horizontal. Dental stone (Modern Materials, Heraeus Kulzer) was then added to the pot and allowed to harden. Intervertebral disc dimensions were measured by caliper and 3D scanning (Structure Sensor, Occipital) to calculate the centre of rotation (posterior 1/3 at mid-disc level). The specimen was then inverted, and the potting procedure repeated. The cephalad pot was then fixed to a stationary beam, while the caudal pot was fixed to a six-axis load cell (MC3A-100, AMTI) mounted to a parallel hexapod robot (R-2000, Mikrolar). Each segment was kept hydrated with gauze soaked in

isotonic saline and covered in plastic wrap during tissue preparation, potting, and testing. Custom software (LabVIEW, National Instruments) was used to test the specimens using an unconstrained force-control system. Force and moment targets were achieved by adjusting the velocity on each axis in proportion to force or moment errors while limiting the maximum velocity of the system. Flexion, extension, lateral bending and axial rotation were performed with three cycles each. Testing velocity was 0.1 degree per second with a maximal moment of 2.0 Nm. The resulting angle vs. moment data were then determined [22].

Supplementary Table 1. Scoring criteria for rodent IVD-NP histology using Safranin O stained tissue sections.

Score	Morphology	Cellularity
**1**	Round, comprising at least half of the disc area	>80% cellularity i.e. rich in notochordal cells.
**2**	Round or irregularly shaped comprising one quarter to half of the disc area	50-80% cellularity with ECM
**3**	Irregularly shaped comprising less than one quarter of the disc area.	10-50% cellularity with cell clusters separated by dense areas of proteoglycan rich ECM.
**4**	NP absent / very small due to hyper-proliferative AF.	Fibrocartilaginous (FC)-NP with <10% cellularity

Supplementary Table 2. Scoring criteria for CD-canine IVD-NP histology using (H&E / Safranin O) stained tissue sections.

Score	Cellularity
**0**	No proliferation
**1**	Increased chondrocyte-like cell density
**2**	Connection of two chondrocytes
**3**	Small size clones (i.e., several chondrocytes group together, i.e. 2–7 cells)
**4**	Moderate size clones (i.e. >8 cells)
**5**	Huge clones (i.e. >15 cells)
**6**	Scar/tissue defects

Supplementary Table 3. Scoring criteria for Safranin O staining intensity

Score	% Intensity
**1**	Mild / Faint
**2**	Moderate
**3**	Strong

Supplementary Table 4. Scoring criteria for immunohistochemistry (IHC)

Score	% Positivity	% Intensity
**0**	None	None
**1**	1 - 10%	Mild / Faint
**2**	10 - 50%	Moderate
**3**	>50%	Strong

Figure legends

Supplementary Figure 1. Panels represent histograms showing average values of (**a**) % cell viability (48 hrs and 72 hrs); (**b**) % cell proliferation (72 hrs) and (**c**) fold change in expression of aggrecan, Col2A1 and HAPLN1 mRNA levels in human NP cells. Each histogram shows mean±SD, *p≤0.05.

Supplementary Figure 2. Representative image showing 3T MRI of CD-canines (B4, that received PBS injection) and (B1 and B2, that received NTG-101 injection) lumbar spine with well-hydrated discs prior to injury (baseline MRI) and the corresponding scans performed 14 weeks post treatment. MR) scans revealed no significant differences in IVDs 14 weeks post-treatment from the baseline scans.

Supplementary Figure 3. (**a**) Canines were anaesthetized prior to preparation for asceotic injection and affixed within radiographically transparent support frame (white arrows); (**b**) Representative image of Dicom imaging software disc height measurements. Note bottom right corner displays calibration measurement; (**c**) Histograms showing mean disc height of untreated discs adjacent to either PBS or NTG-101 treated IVD levels.

Supplementary Figure 4. Panels represent histograms showing average values of scores for (**a**) Cellularity(C); (**b**) Safranin O staining intensity (I). Each histogram shows mean±SD, *p≤0.05 for PBS vs. NTC. Panels presenting histograms showing fold change in expression of (**c**) aggrecan, (**d**) Col2A1, (**e**) IL-6 and (**f**) IL-8 mRNA levels in beagle NP tissues in healthy, no treatment controls (NTC) and injured IVDs that received an intra-discal injection of either vehicle (PBS, 1X) or NTG-101 as determined using quantitative real time - PCR. Each histogram shows mean±SD, *p≤0.05 for NTG-101 vs. PBS.

Supplementary Figure 1.

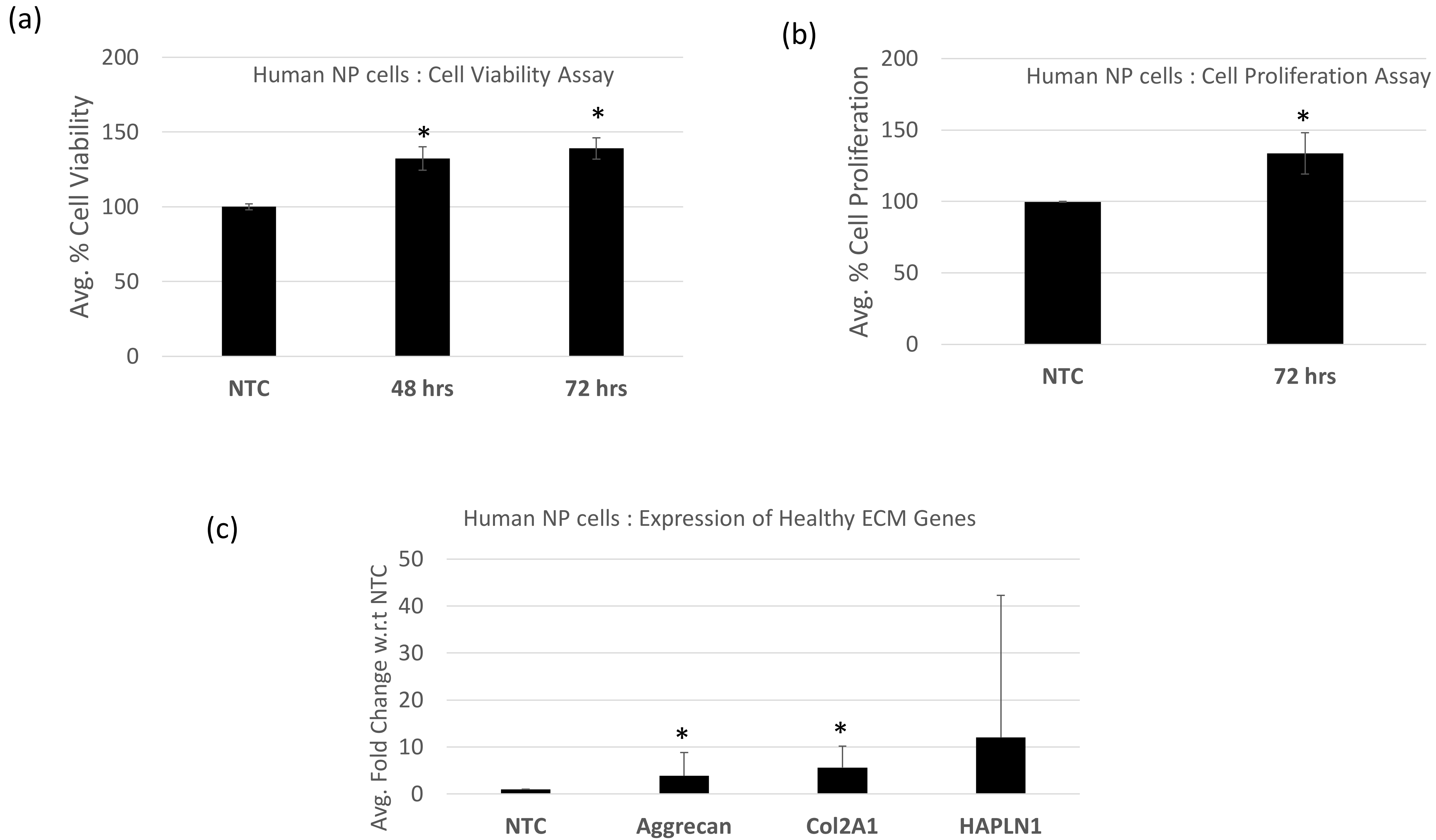

Supplementary Figure 2.

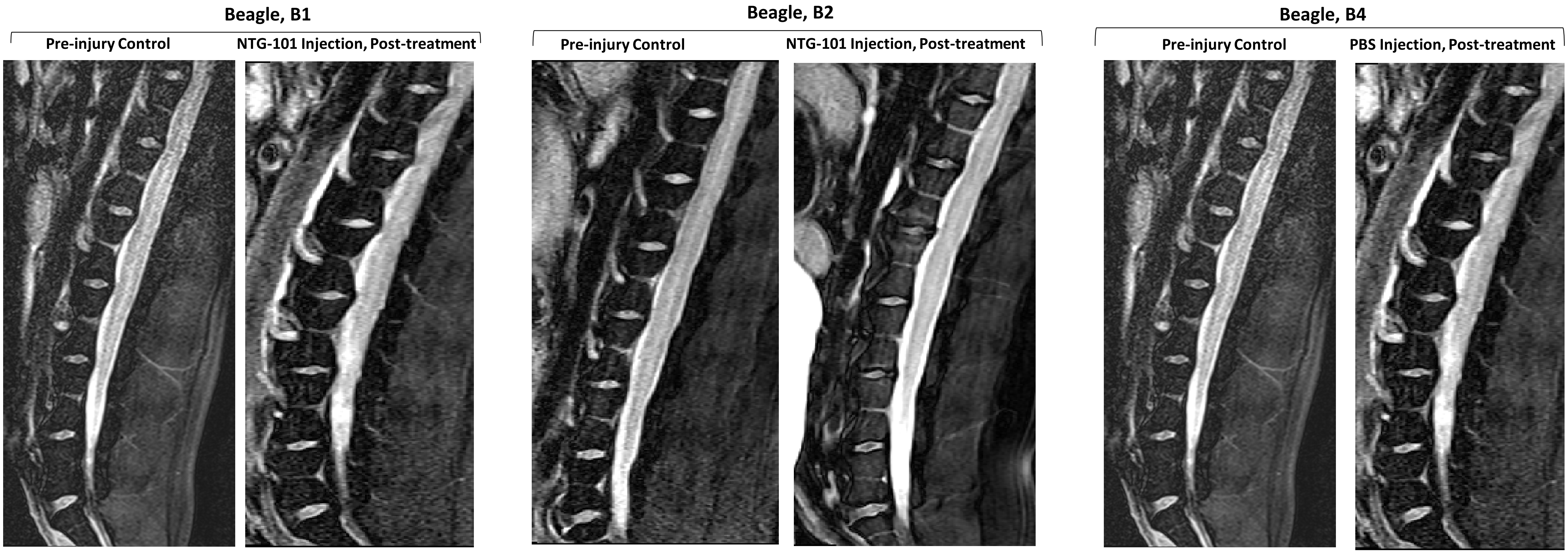

Supplementary Figure 3.

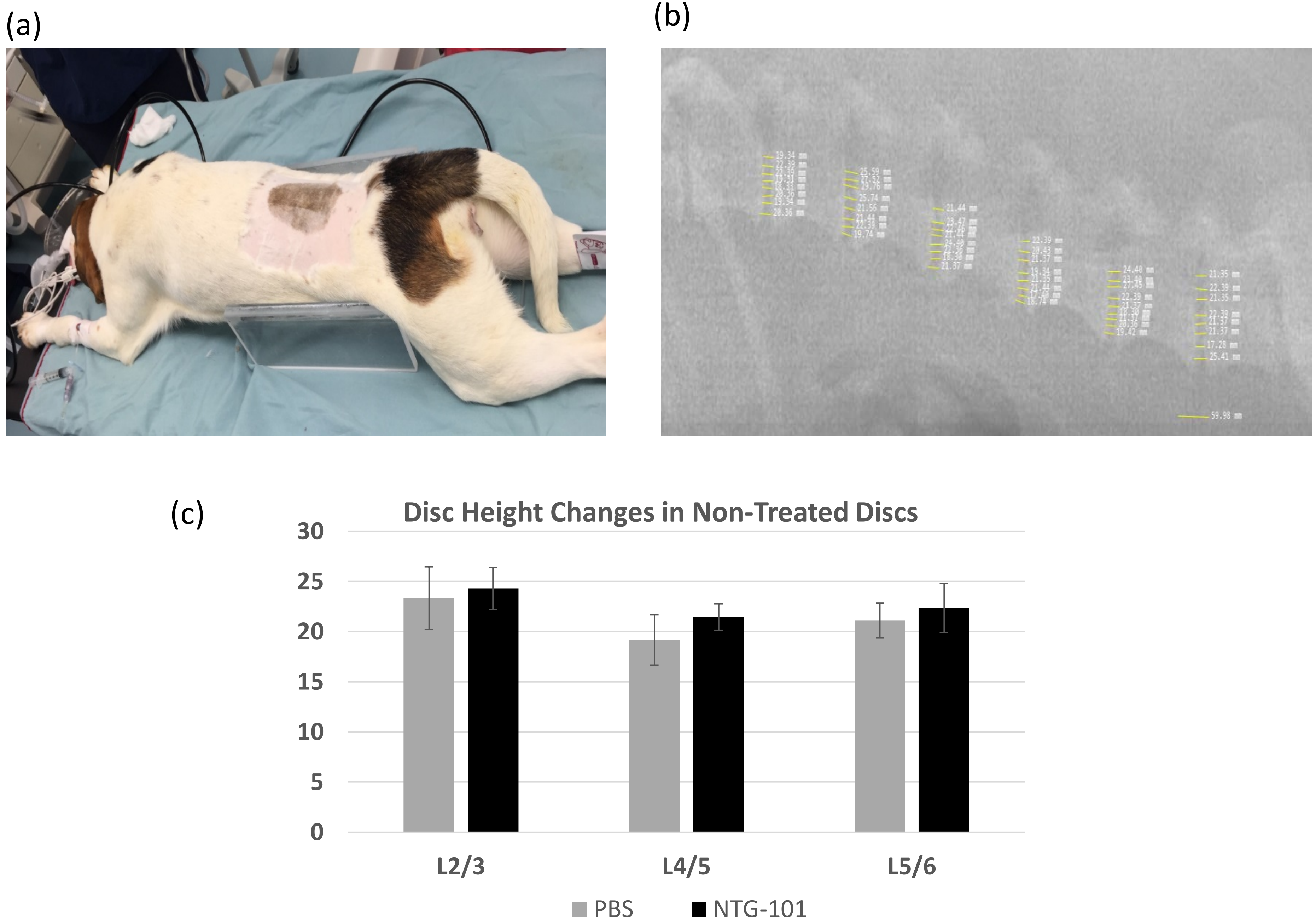

Supplementary Figure 4.

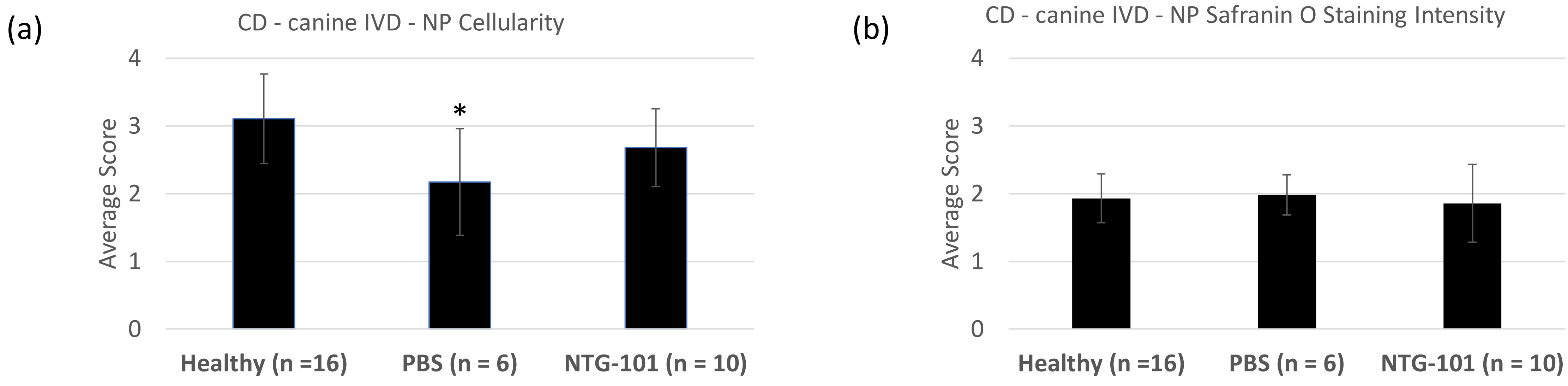

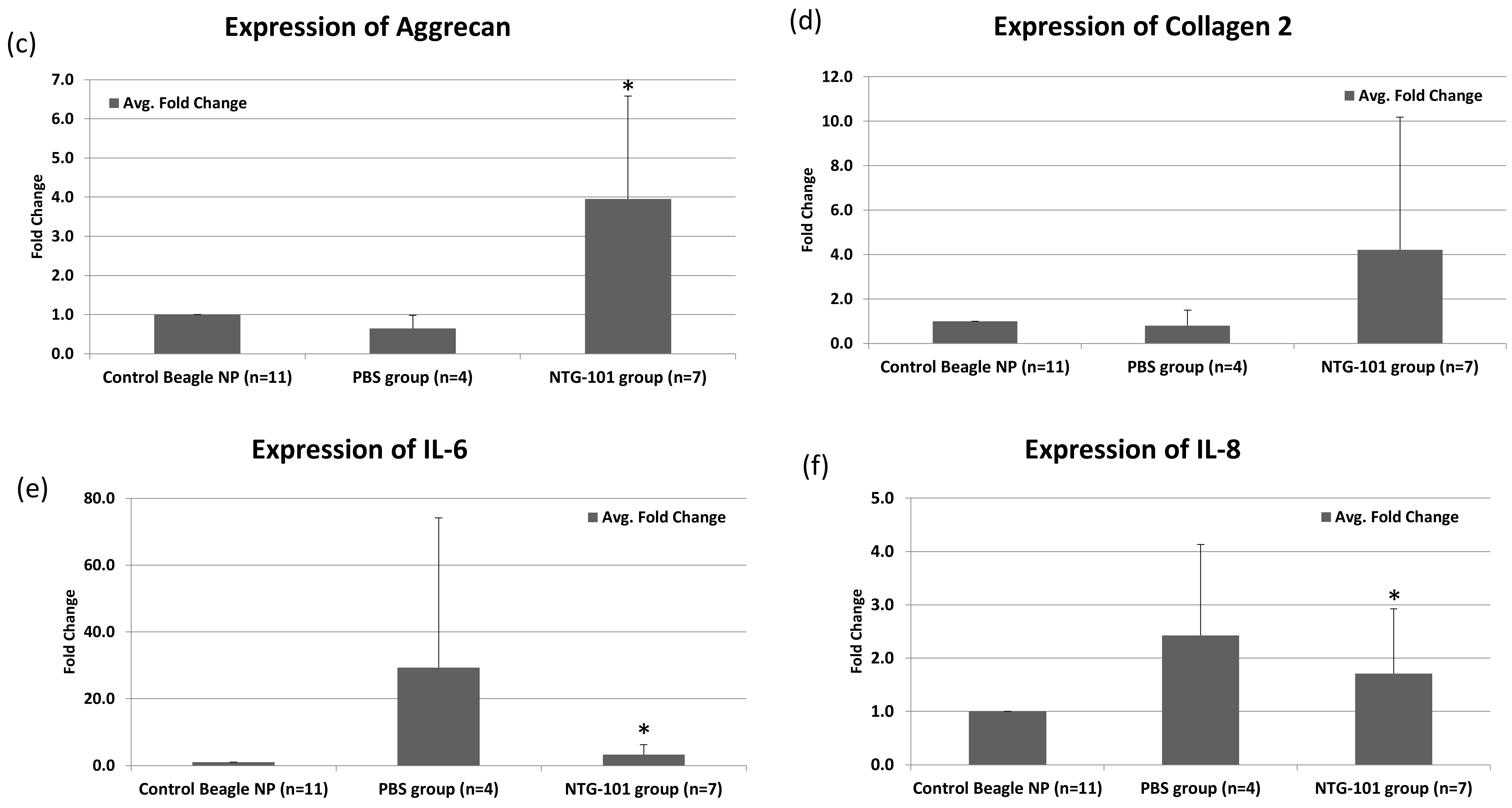
(c)
Expression of Aggrecan
Avg. Fold Change
Fold Change
0.0
1.0
2.0
3.0
4.0
5.0
6.0
7.0
*
Control Beagle NP (n=11)
PBS group (n=4)
NTG-101 group (n=7)
(d)
Expression of Collagen 2
Avg. Fold Change
Fold Change
0.0
2.0
4.0
6.0
8.0
10.0
12.0
Control Beagle NP (n=11)
PBS group (n=4)
NTG-101 group (n=7)
(e)
Expression of IL-6
Avg. Fold Change
Fold Change
0.0
20.0
40.0
60.0
80.0
*
Control Beagle NP (n=11)
PBS group (n=4)
NTG-101 group (n=7)
(f)
Expression of IL-8
Avg. Fold Change
Fold Change
0.0
1.0
2.0
3.0
4.0
5.0
*
Control Beagle NP (n=11)
PBS group (n=4)
NTG-101 group (n=7)
